# A detrimental role of RelB in mature oligodendrocytes during experimental acute encephalomyelitis

**DOI:** 10.1186/s12974-019-1548-7

**Published:** 2019-07-30

**Authors:** Angela S. Gupta, Debolina D. Biswas, La Shardai N. Brown, Karli Mockenhaupt, Michael Marone, Andrew Hoskins, Ulrich Siebenlist, Tomasz Kordula

**Affiliations:** 1 Department of Biochemistry and Molecular Biology, Virginia Commonwealth University, School of Medicine and the Massey Cancer Center, Richmond, VA 23298 USA; 2 Laboratory of Molecular Immunology, National Institutes of Allergy and Infectious Diseases, National Institutes of Health, Bethesda, MD 20892 USA

**Keywords:** EAE, Inflammation, RelB, Oligodendrocytes, Astrocytes, NF-κB

## Abstract

**Background:**

Multiple sclerosis (MS) is an autoimmune demyelinating disease of the central nervous system (CNS). It is firmly established that overactivation of the p65 (RelA) nuclear factor kappa B (NF-κB) transcription factor upregulates expression of inflammatory mediators in both immune and non-immune resident CNS cells and promotes inflammation during MS. In contrast to p65, NF-κB family member RelB regulates immune cell development and can limit inflammation. Although RelB expression is induced during inflammation in the CNS, its role in MS remains unknown.

**Methods:**

To examine the role of RelB in non-immune CNS cells, we generated mice with RelB specifically deleted in astrocytes (RelB^ΔAST^), oligodendrocytes (RelB^ΔOLIGO^), or neural progenitor-derived cells (RelB^ΔNP^). We used experimental autoimmune encephalomyelitis (EAE), an accepted mouse model of MS, to assess the effect of RelB deletion on disease outcomes and performed analysis on the histological, cellular, and molecular level.

**Results:**

Despite being a negative regulator of inflammation, conditional knockout of RelB in non-immune resident CNS cells surprisingly decreased the severity of EAE. This protective effect was recapitulated by conditional deletion of RelB in oligodendrocytes but not astrocytes. Deletion of RelB in oligodendrocytes reduced disease severity, promoted survival of mature oligodendrocytes, and correlated with increased activation of p65 NF-κB.

**Conclusions:**

These findings suggest that RelB fine tunes inflammation and cell death/survival during EAE. Importantly, our data points out the detrimental role RelB plays in controlling survival of mature oligodendrocytes, which could be explored as a viable option to treat MS in the future.

**Electronic supplementary material:**

The online version of this article (10.1186/s12974-019-1548-7) contains supplementary material, which is available to authorized users.

## Background

Multiple sclerosis (MS) is a chronic inflammatory autoimmune demyelinating disease of the central nervous system (CNS) that manifests with symptoms such as muscle weakness, impaired motor skills and coordination, and sensory loss. Although the primary cause of the disease is not known, demyelination, primarily mediated by T cells that are reactive to myelin antigens, and ongoing inflammation are believed to cause damage to the CNS. MS lesions are commonly characterized by apoptotic loss of oligodendrocytes, loss of myelin-associated glycoproteins, immune cell infiltration, activation of resident glial cells, and axon degeneration [1, 2].

Ubiquitously expressed transcription factors of the nuclear factor kappa B (NF-κB) family regulate many cellular processes, including cell proliferation and survival, production of inflammatory mediators, differentiation of T cells, and maturation of dendritic cells [3, 4]. NF-κB family consists of five members: p65 (RelA), RelB, c-Rel, p50/p105, and p52/p100 that can form a variety of homo- and heterodimers. Under resting conditions, NF-κB proteins are sequestered in the cytoplasm to prevent aberrant activation. A typical inflammatory stimulus activates the canonical NF-κB pathway triggering degradation of inhibitory proteins, such as inhibitor of NF-κB alpha (IκBα), translocation of p65/p50 heterodimers to the nucleus, and induction of p65/p50-dependent genes [5]. It is now firmly established that overactivation of p65 in immune cells contributes to inflammation in MS. There is an increased nuclear localization of p65 in microglia and macrophages in active lesions of human MS patients [6]. Accordingly, an increased p65 DNA binding has been reported in rat spinal cords during experimental autoimmune encephalomyelitis (EAE), which is a rodent model of MS [6, 7]. Furthermore, microarray studies have demonstrated increased expression of NF-κB proteins in MS patients [8]. Conversely, preventing degradation of IκBα decreases the incidence and severity of EAE by decreasing antigen-specific T cell responses [9]. c-Rel, which is a target of the canonical NF-κB pathway, is also crucial for the development of EAE, and c-Rel knockout mice are completely protected from the disease. c-Rel knockout mice are characterized by decreased numbers of Th17 and Th1 autoreactive T cells [10].

In contrast to p65, RelB is mostly known as a target of the non-canonical NF-κB pathway, which is triggered by specific ligands that control development of lymphoid tissues [11]. These ligands induce processing of p100 to p52 and subsequent translocation of RelB/p52 heterodimers to the nucleus [12]. Interestingly, in the presence of high RelB expression [13], RelB/p50/IκBα complexes form and are activated by the canonical pathway [13, 14], but this is limited since RelB has higher affinity for p100/p52 than for p50 [15]. The crucial function of RelB in regulating immunity is best exemplified through knockout animal models. RelB knockout mice have a shorter lifespan and severe autoimmunity [11, 16, 17]. Because RelB plays a crucial role in immune cell development, particularly dendritic cells, RelB knockout mice lack Peyer’s patches, germinal centers, and dendritic cell networks [12, 18]. Furthermore, its loss results in a lack of negative T cell selection, and thus severe multiorgan inflammation with both T cell and monocytic infiltrates [19]. Nevertheless, RelB can also repress p65 activity through multiple mechanisms [20–24], which provides a negative feedback loop suppressing cytokine and chemokine expression. Indeed, RelB has been implicated in establishing tolerance in macrophages during septic shock [22]. We have recently shown that RelB also limits cytokine expression in astrocytes, which limits neuroinflammation [24]. Although RelB plays a crucial role in inflammation and immune cell development, its role has not been studied in animal models of MS.

While peripheral and local immune cells are central to the pathogenesis of MS, it is now evident that resident non-immune CNS cells are also critical. Indeed, activation of astrocytes during MS is an important process that directly contributes to the pathology of the disease [25–27]. NF-κB signaling is highly activated in reactive astrocytes and modulates expression of inflammatory mediators, cell death, and cell survival. Accordingly, overexpression of dominant-negative IκBα in astrocytes, which inhibits canonical NF-κB signaling, decreases EAE severity [28]. Multiple intrinsic signaling cascades also modulate susceptibility of oligodendrocytes to injury and thus disease severity [29–31]. Blocking p65 activation in oligodendrocytes has recently been found to increase oligodendrocyte death and reduce remyelination [32]. Although it is evident that p65 plays an important role in non-immune cells of the CNS during MS, the role of RelB has not been investigated. RelB is expressed at low levels in astrocytes [14, 24], but its expression is increased in astrocytes during EAE [21]. Since RelB suppresses cytokine expression in astrocytes and thus regulates neuroinflammation [24], we asked whether RelB in non-immune CNS cells modulates severity of EAE. Understanding the role of RelB in the pathogenesis of MS could provide clues for future therapeutic approaches.

## Materials and methods

### Mice

Mice with the RelB allele flanked by loxP sites generated by Dr. Ulrich Sibienlist (NIH) were bred with GFAP-Cre (Jackson Laboratory) to generate RelB astrocyte-specific conditional knockout mice (RelB^ΔAST^), nestin-Cre (Jackson Laboratory) to generate RelB neural progenitor-specific conditional knockout mice (RelB^ΔNP^), and CNPase-Cre (from Dr. X. Li, Cleveland Clinic, Cleveland, OH) to generate RelB oligodendrocyte-specific knockout mice (RelB^ΔOLIGO^). Mice were housed at Virginia Commonwealth University according to guidelines of the Institutional Animal Care and Use Committee. The mouse protocols were approved by the Institutional Animal Care and Use Committee. Animals were housed in the animal facility, with a 12-h light/dark cycle, and provided water and standard laboratory chow ad libitum. Randomly chosen littermates (males and females) were used for all experiments. All animals were included for data analysis unless they reached a set humane endpoint (20% weight loss) before the end of experiment. The group sizes for each experiment are provided in figure legends. The disease progress was recorded for all experimental animals, while molecular analysis was performed in smaller animal groups that were analyzed using statistics. To establish statistical significance, data were analyzed by ANOVA (multiple comparisons) and both F and *p* values are indicated in the figure legends. Furthermore, post-hoc Sidak’s test was used for these multiple comparisons (*p* values from Sidak’s tests are also indicated). For comparisons of two groups, data were analyzed by *T* test and both *F* and *p* values are indicated in the figure legends. Asterisk designates statistical significance with both *F* and *p* values indicated.

### Experimental autoimmune encephalomyelitis

Each mouse received subcutaneous 200 μg MOG35–55 peptide (AnaSpec) emulsified in CFA containing 500 μg *Mycobacterium tuberculosis* H37Ra (Difco, Detroit, MI) and intraperitoneal 200 ng pertussis toxin (Enzo Life Sciences, Farmingdale, NY). A booster dose of 200 ng pertussis toxin was administered 2 days after immunization. Mice were clinically scored and weighed daily, and the severity of the disease was quantified using a five-point scale: 0, no symptoms; 1, limp tail; 2, limp tail with loss of righting; 3, paralysis of single hind limb; 4, paralysis of both hind limbs; and 5, death. Two-three independent experiments were performed (as indicated in figure legends), and cumulative data are presented. Tissues were collected at day 15 from PBS- and MOG-injected animals for molecular/cellular analysis.

### Cell culture

To prepare mouse cortical astrocytes, cerebral cortices were aseptically dissected and meninges were removed. Tissue was mechanically dissociated, incubated with trypsin and DNaseI at 37 °C for 30 min, and centrifuged. Tissue was filtered through a 70 μm filter and re-centrifuged. Cells were resuspended and plated in dishes pre-coated with poly-d-lysine. Cells were cultured in Dulbecco’s modified Eagle’s medium supplemented with 10% fetal bovine serum, penicillin/streptomycin, sodium pyruvate, and non-essential amino acids.

### Western blotting

Cells or ground flash frozen tissue were lysed in 10 mM Tris (pH 7.4), 150 mM sodium chloride, 1 mM EDTA, 1% Nonidet P-40, 1% Triton X-100, 1 mM sodium orthovanadate, 0.2 mM PMSF, and Pierce protease inhibitor mixture. Samples were separated on a 10% gel and transferred onto nitrocellulose membranes (GE Healthcare). Anti-β-tubulin (sc-9104), anti-RelB (sc-226), anti-p65 (sc-372) antibodies (Santa Cruz Biotechnology); anti-GAPDH (5174); and anti-phospho-p65(S536) (3031) antibodies (Cell Signaling) were used. Antigen-antibody complexes were visualized by enhanced chemiluminescence using Immobilon Western blotting kit (Millipore).

### Quantitative PCR

Total RNA was prepared from flash frozen tissue with Trizol (Life Technologies), reverse transcribed with the high-capacity cDNA kit (Applied Biosystems), and amplified on the BioRad CFXConnect Real-time System. SYBR Green intron-spanning pre-design qPCR primers (BioRad) were used. Gene expression levels were normalized to GAPDH and represented as fold expression over control.

### Immunofluorescence

Animals were perfused with 4% paraformaldehyde, tissue was embedded in optimal cutting medium, and 40 μm frozen sections were prepared. For fluoromyelin staining, slides were rehydrated in PBS for 20 min and then flooded with 1:300 diluted stain for 20 min. Counterstaining was done using Hoechst stain for 5 min, and slides were mounted using vectashield mounting medium (Vector Laboratories). Slides were imaged using the Zeiss LSM 700. For antibody staining, sections were incubated with primary anti-RelB, anti-GFAP, anti-p65, anti-phospho-p65 (all 1:300, Cell Signaling), anti-CC1 (1:200, Millipore), or anti-Iba1 (1:500, Wako) antibodies overnight at 4 °C. Subsequently, sections were incubated with Alexa Fluor 488 or Alexa Fluor 594 secondary antibodies (1:500, Invitrogen) for 1 h at room temperature. Slides were mounted and imaged as described above. No fluorescence crossover was found between the channels, and images were collected separately using the appropriate laser excitation. Images were analyzed using ImageJ.

### Immunohistochemistry

Animals were perfused with 4% paraformaldehyde and tissue was paraffin-embedded, sectioned, and H&E-stained at the Cancer Mouse Models Core Facility (VCU, Richmond, VA). Slides were imaged using the Zeiss AxioImager A1 as indicated. Infiltration of immune cells was quantified by counting immune cells in the peripheral regions of the spinal cords.

### Isolation and analysis of immune cells in the CNS

Brains from the mice were dissociated using Wheaton Dounce glass tissue grinders, strained through 70 μm filter, and subjected for centrifugation at 1500 rpm for 5 min at 4 °C. Pelleted cells were resuspended in 10 ml of 30% Percoll (Amersham Bioscience) and centrifuged onto a 70% Percoll for 30 min at 2600 rpm. Cells were collected at the 30–70% interface and stained with fluorescence-conjugated monoclonal antibodies against CD45 (clone 30-F11), CD11b (clone M1/70), CD4 (clone GK 1.5), CD8 (clone 53–6.7), and isotype control antibodies (Biolegend) were used to analyze cells.

### Statistical analysis

Statistical analysis was performed using GraphPad Prism 7. Values are displayed as mean ± standard error. *T* tests and ANOVAs were performed as indicated. Post-hoc Sidak’s was used for multiple comparisons.

## Results

### Deletion of RelB in non-immune CNS cells reduces severity of EAE

**Fig. 1:**
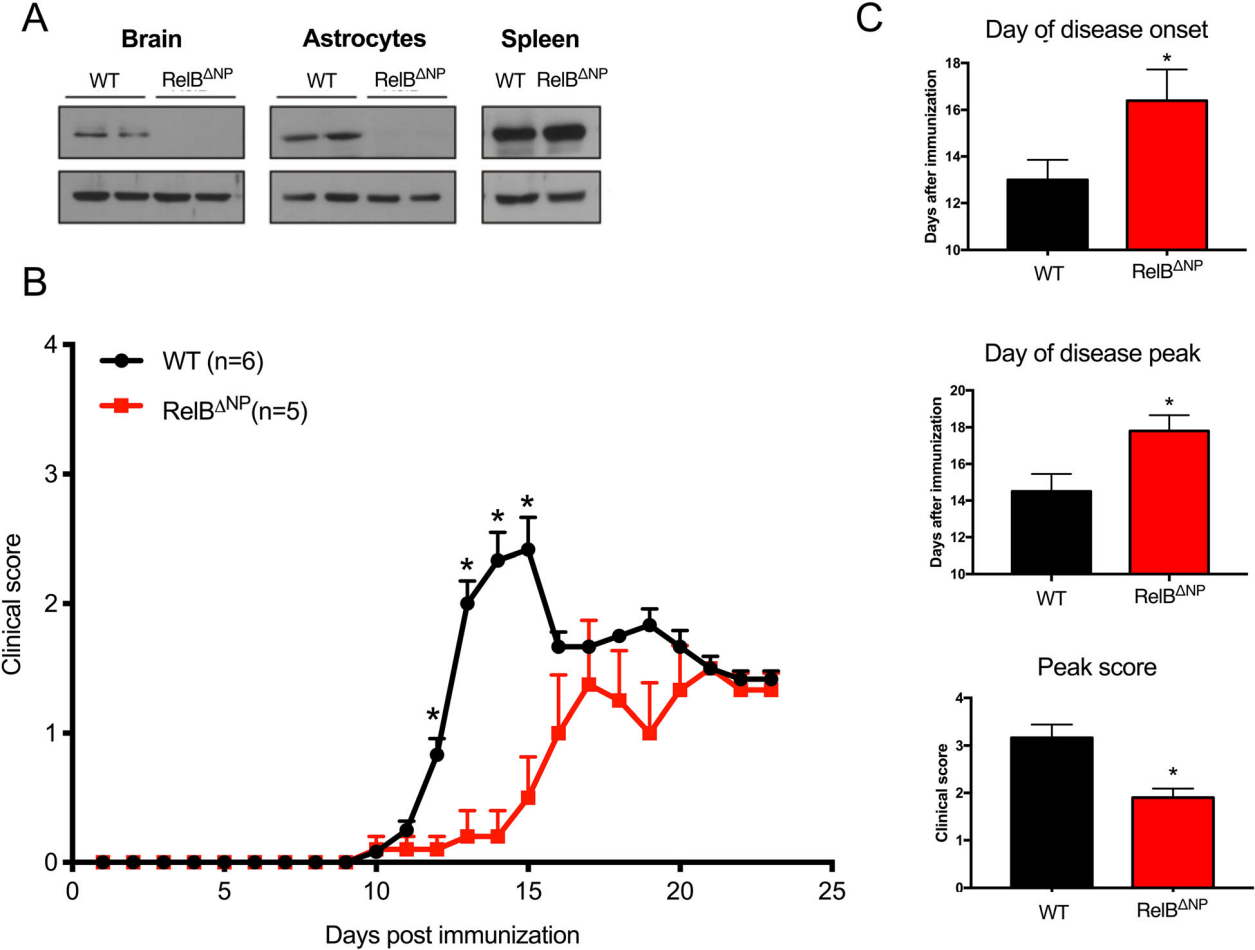
Ablation of RelB in non-immune CNS cells reduces severity of experimental autoimmune encephalomyelitis. **a** Deletion of RelB was verified by western blotting in the brains, cultured mouse astrocytes, and spleens from WT (RelB^loxP/loxP^) and RelB^ΔNP^ mice. **b**, **c** EAE was induced and clinical scores were recorded for 23 days. *n* = 5–6 mice per group. **b** Multiple *T* tests (*p < 0.05). **c** *T* tests: day of onset (*F* = 4.54, *p* = 0.038); day of peak (*F* = 11.50, *p* = 0.036); peak score (*F* = 13.20, *p* = 0.047)

**Fig. 2:**
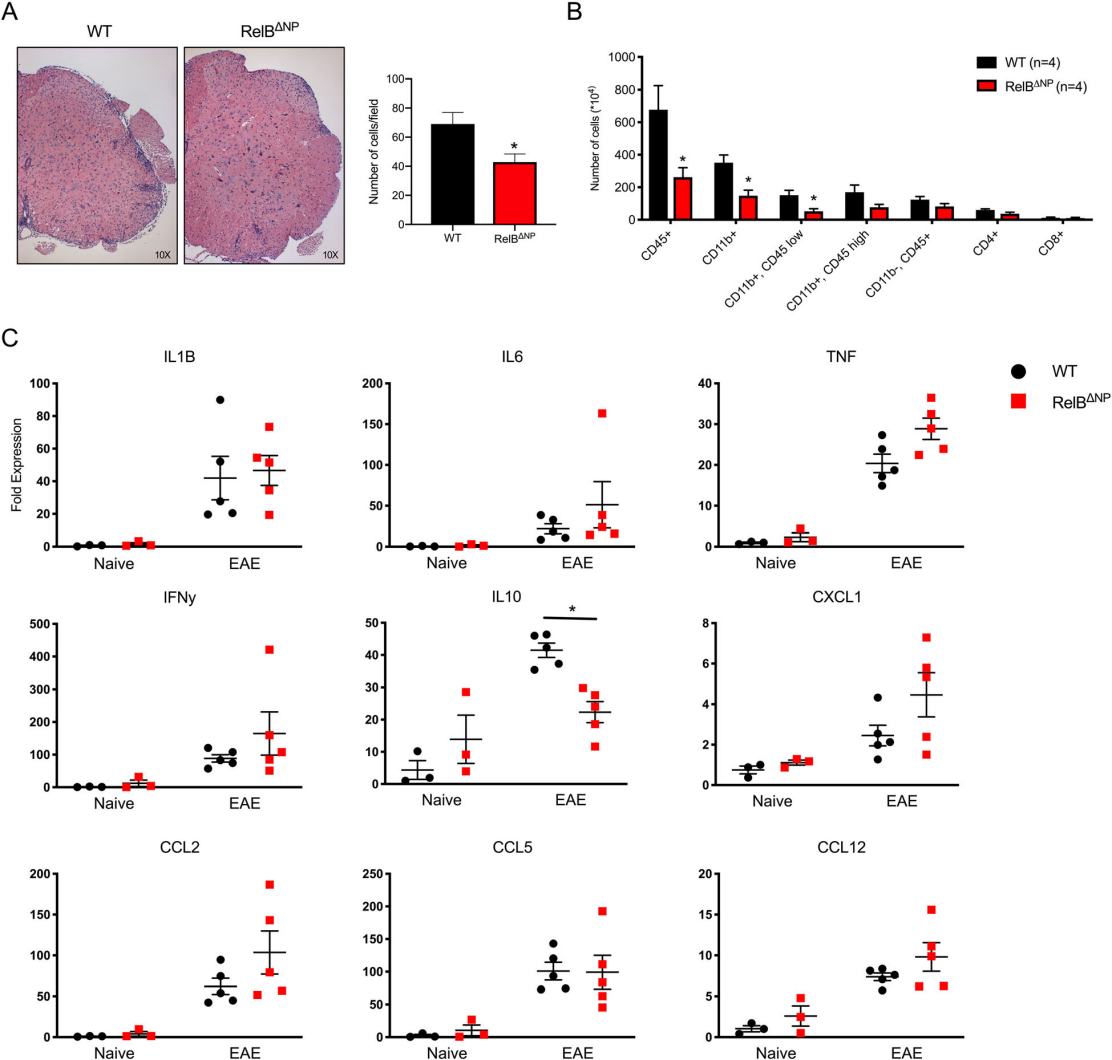
Decreased immune cell infiltration during EAE in RelB^ΔNP^ mice. EAE was induced in WT (RelB^loxP/loxP^) and RelB^ΔNP^ mice. Tissues were collected at the peak of disease. **a** Lumbar spinal cords (L2–L4) (left panels) were stained with hematoxylin and eosin. Quantification of immune cells infiltration into peripheral regions of spinal cords. *n* = 6, *T* test: *F* = 2.024, *p* = 0.0226). **b** Flow cytometry was conducted to quantify the indicated cells in the brains. *n* = 4 mice per group. *T* tests: CD45 (*F* = 6.486, *p* = 0.041); Cd11b^+^ (*F* = 1.956, *p* = 0.014); CD11b^+^CD45^low^ (*F* = 3.497, *p* = 0.026). **c** Expression of the cytokines was examined by qPCR in lumbar spinal cords. *n* = 3–5 mice per group. IL-10, two-way ANOVA (*F* = 12.84, *p* = 0.004), Sidak’s test (*p* = 0.005)

Since RelB is upregulated in astrocytes during both EAE [33] and experimental LPS-induced neuroinflammation [24] and limits astrocytic cytokine production [24], we predicted that it may play important functions in non-immune CNS cells during EAE, and its deletion may exaggerate severity of the disease. To test this hypothesis, we crossed nestin-Cre mice with RelB^loxp/loxp^ mice to generate nervous system-restricted RelB conditional knockout mice deficient of RelB in astrocytes, oligodendrocytes, neurons, and adult neural progenitors (Fig. 1a). These mice were born with expected Mendelian ratios (50% WT (RelB^loxp/loxp^) and 50% RelB^ΔNP^ (nestin-Cre; RelB^loxp/loxp^), were phenotypically normal, and displayed no obvious inflammation or neurological deficits. Surprisingly, RelB^ΔNP^ mice had reduced severity of EAE both at initiation and throughout the entire course of the disease (Fig. 1b, Additional file 1: Figure S1). These mice manifested EAE at a later day of onset, demonstrated a later day of disease peak, and had a lower peak score compared to WT littermates (Fig. 1c). Although nestin-Cre driver mice display metabolic phenotype [34], the unexpected reduced severity of the EAE in RelB^ΔNP^ mice was a result of RelB deletion since EAE severity was comparable in RelB^loxp/loxp^ mice and nestin-Cre; RelB^WT/WT^ mice (Additional file 1: Figure S2). In agreement with lower clinical scores, the RelB^ΔNP^ mice also had decreased immune cell infiltration in the white matter of the lumbar spinal cord, particularly along the ventral tracts (Fig. 2a). Decreased meningeal inflammation was also evident. Furthermore, flow cytometry analysis indicated decreased numbers of brain-infiltrating immune cells, including CD11b^+^ myeloid cells (both CD11b^+^ CD45^low^ microglia and CD11b^+^ CD45^high^ monocytes), and also CD4^+^ and CD8^+^ T cells (Fig. 2b, Additional file 1: Table S1). Since RelB negatively regulates cytokine expression in many cell types [35], including astrocytes [24], we examined cytokine mRNA levels in the spinal cords during EAE. RelB^ΔNP^ mice expressed anti-inflammatory IL-10 at lower level (Fig. 2c); however, expression of many proinflammatory cytokine and chemokine mRNAs were only incrementally increased in RelB^ΔNP^ mice in comparison to RelB^loxp/loxp^ littermates. Thus, although deletion of RelB in the non-immune CNS cells slightly enhances cytokine expression during EAE, paradoxically RelB in these cells is protective.

### Astrocytic RelB has a limited effect on the severity of EAE

**Fig. 3:**
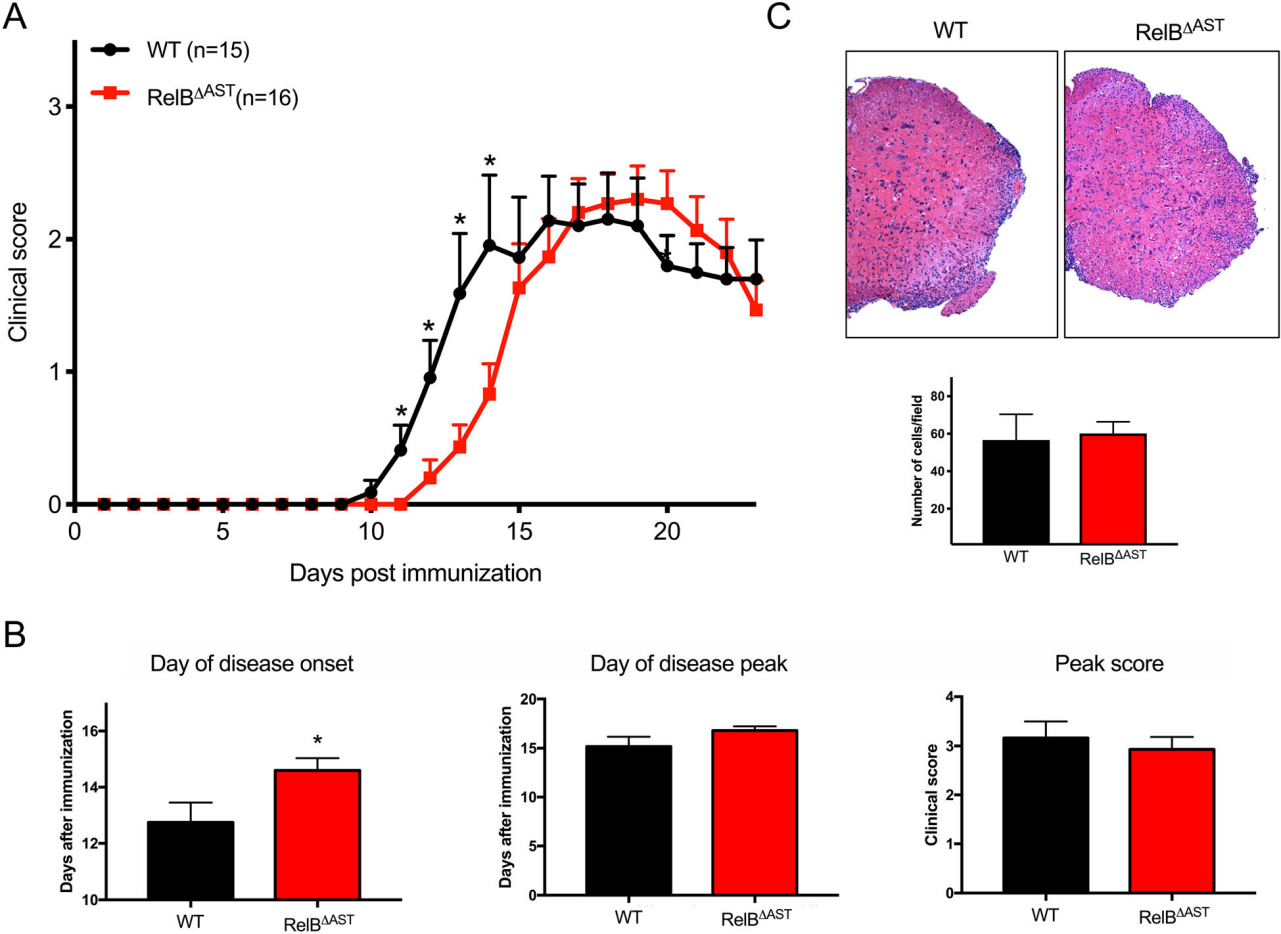
Astrocyte-restricted ablation of RelB delays the onset of EAE. EAE was induced in WT (RelB^loxP/loxP^) and RelB^ΔAST^ mice. **a**, **b** Clinical scores were recorded for 24 days. *n* = 15–16 mice per group. **a** Multiple *T* tests (*p < 0.05). **b** *T* tests: day of onset (*F* = 4.274, *p* = 0.013); day of peak (*F* = 2.129, *p* = 0.184); peak score (*F* = 1.390, *p* = 0.554). **c** At the peak of disease, lumbar spinal cords (L2–L4) (left panels) were stained with hematoxylin and eosin. Quantification of immune cells infiltration into peripheral regions of spinal cords. *n* = 6, *T* test: *F* = 4.879, *t* test *p* = 0.824

Since knockout of RelB in non-immune CNS cells reduced severity of EAE, we sought to determine which cell type is contributing to this phenotype. Astrocytes are critical regulators of immune and inflammatory responses in the brain [36], and they upregulate expression of RelB during EAE [33]. To determine if astrocytic RelB regulates the severity of the disease, we used recently generated RelB^ΔAST^ mice [24] and their RelB^loxp/loxp^ littermates in EAE experiments. The RelB^ΔAST^ mice are phenotypically normal and are generated with the expected Mendelian distribution. Although the onset of EAE in RelB^ΔAST^ mice was delayed in comparison to WT littermates (Fig. 3a), the severity of EAE (peak score and day of disease peak) was similar (Fig. 3b). In concordance with the similar disease severity, both RelB^ΔAST^ mice and WT littermates demonstrated a similar level of diffuse inflammatory cell infiltrate in the lumbar spinal cord, as well as some focal areas of immune cell infiltration in the white matter (Fig. 3c). We concluded that astrocytic RelB cannot account for the protective phenotype of RelB^ΔNP^ mice, suggesting that RelB plays critical protective role in another cell type.

### Deletion of RelB in oligodendrocytes attenuates EAE disease severity

**Fig. 4:**
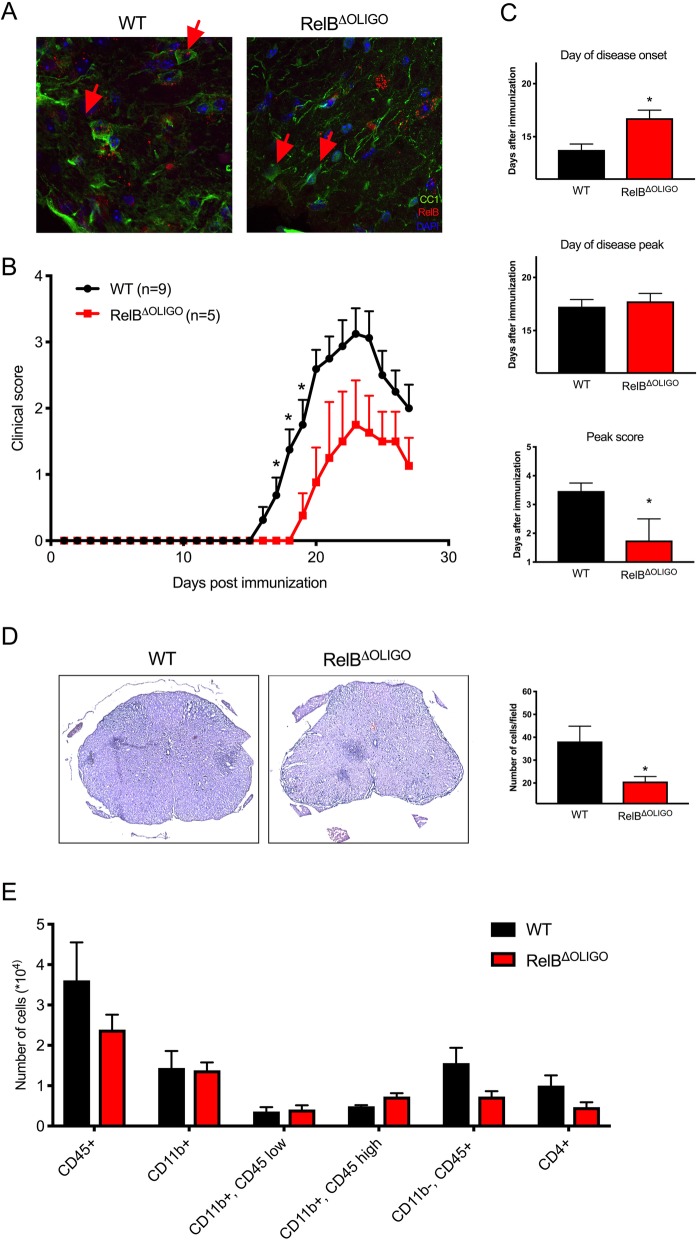
Deletion of RelB in mature oligodendrocytes reduces the severity of EAE. **a** RelB was visualized in CC1-positive oligodendrocytes in cortical brain sections from WT (RelB^loxP/loxP^) and RelB^ΔOLIGO^ mice by IF. Anti-CC1 and anti-RelB antibodies were used, and sections were counterstained with Hoechst. **b**–**e** EAE was induced in WT and RelB^ΔOLIGO^ mice. **b**, **c** Clinical scores were recorded for 23 days. *n* = 5–9 mice per group. **b** Multiple *T* tests (*p < 0.05). **c** *T* tests: day of onset (*F* = 1.11, *p* = 0.01); day of peak (*F* = 1.619, *p* = 0.65); peak score (*F* = 3.659, *p* = 0.02). **d** At the peak of disease, lumbar spinal cords were stained with hematoxylin and eosin (L2–L4) (left panels). Quantification of immune cells infiltration into peripheral regions of spinal cords. *n* = 6, *T* test: *F* = 9.212, *p* = 0.0321. **e** Flow cytometry was conducted to quantify the indicated cells in the brains. *n* = 3–4 mice per group, *T* tests

Because death of oligodendrocytes is a hallmark of both MS and EAE [37–39], we asked whether deletion of RelB in oligodendrocytes could explain the reduced EAE severity in RelB^ΔNP^ mice. We generated oligodendrocyte-specific RelB conditional knockout mice (RelB^ΔOLIGO^) by crossing RelB^loxp/loxp^ mice with CNPase-Cre driver mice. The RelB^ΔOLIGO^ mice were phenotypically normal and generated progeny as expected. RelB was efficiently deleted from oligodendrocytes since it no longer was detected in CC1-positive cells of RelB^ΔOLIGO^ mice, but it was present in CC1-positive cells of WT littermates (Fig. 4a). Interestingly, induction of EAE in RelB^ΔOLIGO^ mice resulted in reduced severity of the disease in comparison to WT littermates (Fig. 4b). Similar to RelB^ΔNP^ mice, RelB^ΔOLIGO^ mice had a lower peak score and later day of disease onset (Fig. 4c); however, the day of disease peak was not affected. The decreased severity of the EAE in RelB^ΔOLIGO^ mice correlated with a decrease in diffuse inflammatory cell infiltrate throughout the lumbar spinal cord with small scattered focal areas of inflammation (Fig. 4d). There was also a trending decrease in immune cell infiltration in the brain as detected by flow cytometry, which was particularly evident for CD4^+^ T cells (Fig. 4e).

### Oligodendrocyte-restricted ablation of RelB prevents demyelination and decreases loss of mature oligodendrocytes

**Fig. 5:**
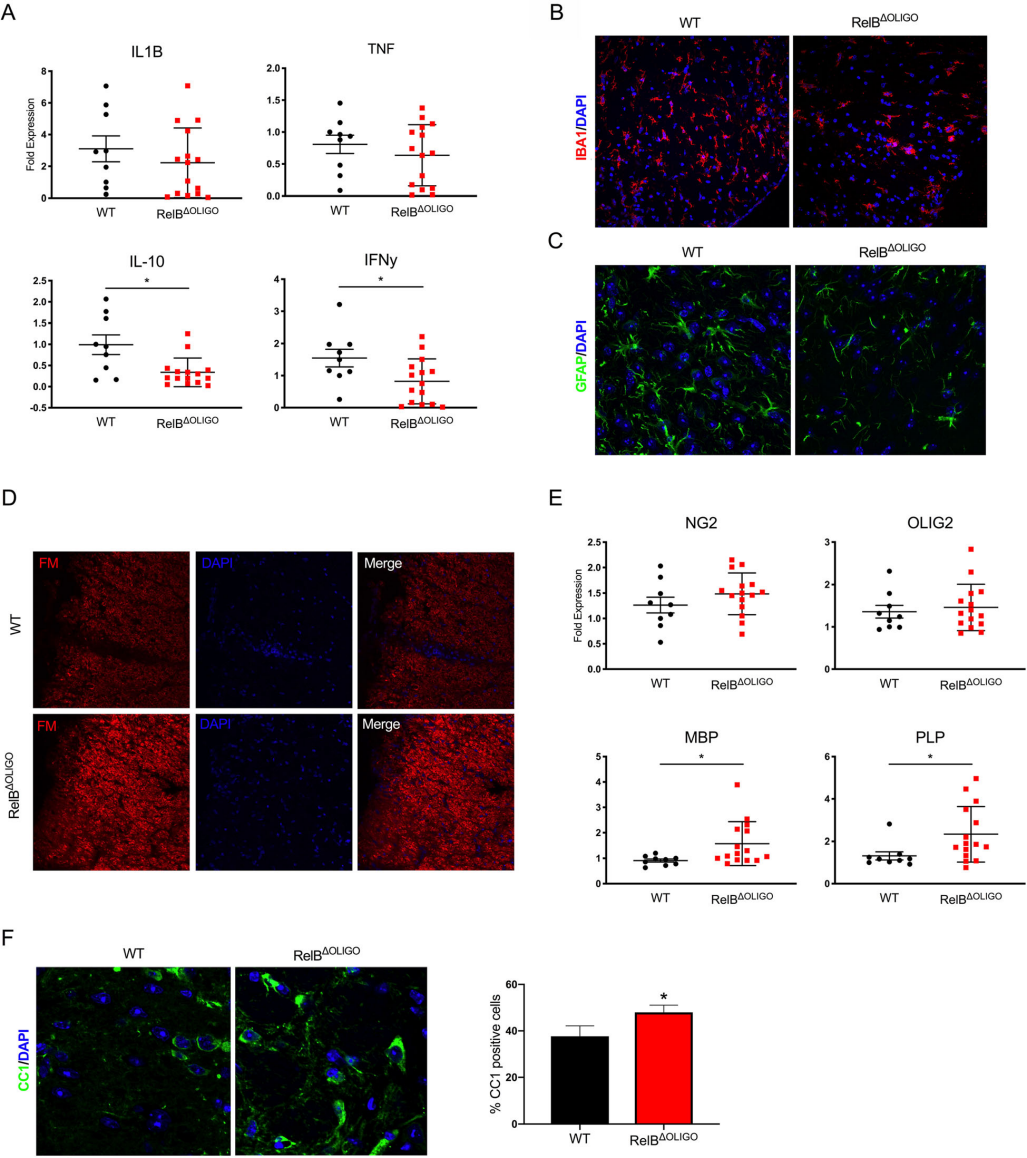
Deletion of RelB in oligodendrocytes prevents loss of mature oligodendrocytes and demyelination. EAE was induced in WT (RelB^loxP/loxP^) and RelB^ΔOLIGO^ mice. Tissues were collected at the peak of disease. **a**, **e** Expression was analyzed by qPCR in lumbar spine tissue. *n* = 8–14 mice per group. *T* tests: IL-10 (*F* = 4.243, *p* = 0.025); IFNg (*F* = 1.384, *p* = 0.032); MBP (*F* = 22.57, *p* = 0.03); PLP (*F* = 5.116, *p* = 0.04). **b**, **c** Immunofluorescence of lumbar spinal cord sections stained with anti-IBA1 (**b**) and anti-GFAP (**c**) antibodies. Sections were counterstained with Hoechst. **d** Lumbar spinal cord sections stained with fluoromyelin and counterstained with Hoechst. **f** Immunofluorescence of lumbar spinal cord sections stained with anti-CC1. Quantification of CC1 positive cells (IF). Images were taken at least two regions from three different animals per genotype. Percentages of CC1-positive cells to nuclei labeled with Hoechst. Measurements were calculated manually using the cell counter tool in ImageJ (*n* = 6–7 per group, *T* test; (*F* = 1.846, *p* = 0.04)

**Fig. 6:**
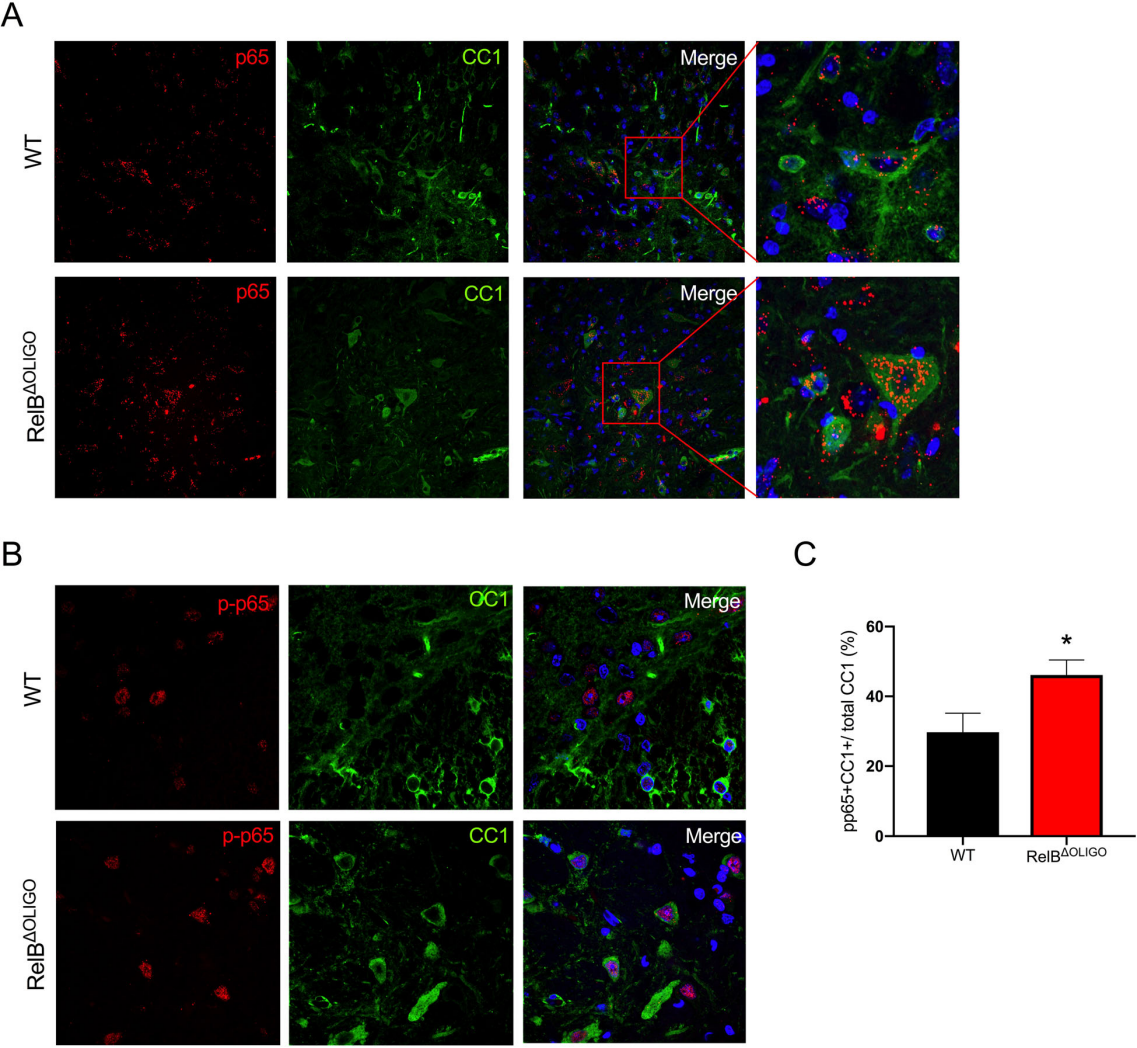
Increased activation of p65 NF-κB in oligodendrocytes of RelB^ΔOLIGO^ mice. EAE was induced in WT (RelB^loxP/loxP^) and RelB^ΔOLIGO^ mice. Tissues were collected at the peak of disease. **a**, **b** Sections of the lumbar spinal cords were stained with anti-p65, anti-pp65, and anti-CC1 antibodies, and counterstained with Hoechst. Higher magnifications are shown (right panels). **c** Quantification of pp65/CC1 double-positive cells (IF) in spinal cords of WT (RelB^loxP/loxP^) and RelB^ΔOLIGO^ mice. Percentages were calculated as the ratio of pp65/CC1 double labeled cells per total CC1 cells measured using the cell counter tool in ImageJ. (*n* = 6–7 per group, *T* test: (*F* = 1.359, *p* = 0.04)

To determine why RelB^ΔOLIGO^ mice develop EAE with reduced severity, we first investigated whether the disease correlates with decreased inflammation characterized by lower expression of cytokines. Activated microglia and reactive astrocytes, but not oligodendrocytes, are the major producers of inflammatory mediators during EAE [40]. Not surprisingly, there was no significant difference between expression of many cytokines and chemokines between RelB^ΔOLIGO^ mice and RelB^loxp/loxp^ littermates (Fig. 5a). Although expression of major proinflammatory cytokine mRNAs, such as IL-1β and TNFα, was comparable, IFNγ and IL-10 mRNA levels were interestingly significantly lower in the spinal cords of RelB^ΔOLIGO^ mice. In concordance with these findings, numbers of both Iba1^+^ myeloid cells (Fig. 5b) and GFAP^+^ reactive astrocytes (Fig. 5c) were significantly diminished in RelB^ΔOLIGO^ mice. Furthermore, RelB^ΔOLIGO^ mice had a higher intensity of myelin staining in the lumbar spinal cords compared to WT littermates (Fig. 5d). Oligodendrocyte death followed by regeneration from oligodendrocyte progenitor cells (OPCs) is characteristic of EAE and is a determinant of the clinical phenotype of paralysis [30, 41]. Since IFNγ is known to induce apoptosis of oligodendrocyte progenitors [42, 43], we examined the expression of markers for OPCs (NG2, OLIG2) and mature oligodendrocytes (MBP, PLP). Although levels of IFNγ were significantly lower in RelB^ΔOLIGO^ mice (Fig. 5a), expression of NG2 and OLIG2 in the lumbar spinal cords were not changed (Fig. 5e), suggesting similar survival of OPCs. In contrast, RelB^ΔOLIGO^ mice were characterized by significantly higher expression of MBP and PLP mRNA in the lumbar spinal cords, indicating that deletion of RelB prevents mature oligodendrocyte loss during EAE (Fig. 5e). Since RelB can inhibit p65 [23, 44, 45] and p65 promotes survival of oligodendrocytes in vitro [46, 47] and during EAE [32], we hypothesized that RelB^ΔOLIGO^ mice were protected due to exaggerated activation of p65. To evaluate this, we first analyzed expression and activation of p65 in CC1-positive mature oligodendrocytes by immunofluorescence. As expected, there was a greater increase in CC1-positive oligodendrocytes in the spinal cords of RelB^ΔOLIGO^ mice than in RelB^loxp/loxp^ littermates (Fig. 5f), which was consistent with the elevated expression of MBP and PLP (Fig. 5e). These CC1-positive oligodendrocytes were also p65-positive (Fig. 6a). Next, we evaluated phosphorylation of p65 on Serine 536 that correlates with p65 activation [48]. We found that activated, phosphorylated p65 was present in the nuclei of CC1-positive oligodendrocytes in both RelB^ΔOLIGO^ mice and their WT littermates (Fig. 6b). Importantly, quantification of these data revealed that the percent of CC1-positive cells that co-stain with p-p65(Ser536) was significantly higher during EAE in RelB^ΔOLIGO^ mice than in WT littermates (Fig. 6c). These data suggest that activation of p65 in CC1-positive oligodendrocytes is exaggerated in the absence of RelB, promoting survival of mature oligodendrocytes and subsequently limiting severity of EAE in RelB^ΔOLIGO^ mice.

## Discussion

RelB has been mostly recognized as a regulator of immune cell development and differentiation that is critical for B cell homeostasis and establishment of lymph nodes and germinal centers [12]. RelB also regulates osteoclast differentiation [49]. These functions of RelB are controlled by the non-canonical NF-κB pathway and RelB/p52 complexes. In contrast, RelB/p50 complexes have recently been identified as critical targets of the necroptosis-independent RIPK3-dependent pathway, promoting inflammation in dendritic cells [50]. Interestingly, oligodendrocyte death involves both necroptosis and RIP3K activation [51, 52]. Over the past decade, RelB also emerged as a critical negative regulator of inflammatory responses in non-lymphoid cells. Multiple mechanisms by which RelB negatively regulates inflammation have been proposed to date, including inhibition of p65 translocation and binding [23], epigenetic silencing of chromatin [22], and dimer switching [44]. RelB also limits production of inflammatory cytokines and curbs inflammatory memory in astrocytes [24]. Indeed, our data confirm that RelB limits cytokine production in non-immune CNS cells during EAE since RelB^ΔNP^ mice have increased proinflammatory and decreased anti-inflammatory cytokine expression. Unexpectedly however, RelB^ΔNP^ mice displayed reduced severity of EAE indicating that RelB plays other important functions that are in fact not related to the control of cytokine production or inflammation. These conclusions are additionally supported by our findings that deletion of RelB in astrocytes, which are known to produce substantial quantities of inflammatory cytokines [53], has limited effect on the severity of EAE. Furthermore, although oligodendrocytes are not known to produce significant amounts of cytokines, deletion of RelB in these cells significantly reduced severity of EAE in RelB^ΔOLIGO^ mice. Thus, the protective effect of RelB deletion in non-immune CNS cells during EAE does not seem to be related to its anti-inflammatory functions but rather non-inflammatory effects in oligodendrocytes. Of note, although we did not find a direct correlation between the severity of EAE and expression of proinflammatory mediators, this can be explained by relatively aggressive disease with high cytokine and chemokine expression in both control and RelB-deficient animals.

We found that RelB exhibits deleterious effects in oligodendrocytes, and these cells are the main targets of autoreactive T cell-mediated response during EAE, which leads to mature oligodendrocyte death, axon degeneration, and increased disease severity [29, 30]. Although deletion of RelB in CNP-expressing oligodendrocytes decreased the amount of IFNγ, known to induce apoptosis of OPCs during EAE [42, 43], it had no effect on the abundance of oligodendrocyte progenitor markers, suggesting similar survival of OPCs. Nevertheless, enhanced differentiation to mature oligodendrocytes in the absence of RelB cannot be fully excluded. Expression of mature oligodendrocyte markers was significantly higher during EAE in RelB^ΔOLIGO^ mice. These results suggest that RelB may limit survival of mature oligodendrocytes, promotes their death during EAE, or suppresses differentiation of OPCs.

Previous studies have demonstrated that the canonical activation of p65/p50 complexes promote oligodendrocyte survival during inflammation [32, 46, 47]. Although the exact mechanism remains elusive, p65 is known to control expression of several anti-apoptotic genes including cIAPs, cFLIP, Bcl-2, Bcl-xL, TRAF1, and TRAF2 [4]. Indeed, we found increased activation of p65 in oligodendrocytes during EAE in RelB^ΔOLIGO^ mice. The increased p65 activation in oligodendrocytes can be explained by the lack of RelB-mediated inhibition that is accomplished by previously described mechanisms [22–24, 44]. However, it is also possible that a proposed p100/IκBδ-mediated crosstalk between canonical and non-canonical NF-κB pathways [54] regulates p65 activation and thus mature oligodendrocyte survival. Since RelB is known to stabilize p100/IκBδ [55], its deletion may limit the amount of p100/IκBδ in oligodendrocytes and thus indirectly remove p100/IκBδ-mediated inhibition of the p65/p50 activation. It is highly probable that all these RelB-dependent mechanisms function in vivo to fine tune inflammation and cell death/survival. Our data suggest that specific targeting of RelB in oligodendrocytes in the future could be explored as a viable option to limit oligodendrocyte loss during MS.

## Conclusions

Overall, our data demonstrate that although RelB suppresses cytokine production by non-immune cells in the CNS, it plays an additional deleterious role in controlling survival of mature oligodendrocytes during EAE. Thus, RelB fine tunes inflammation and cell death/survival during EAE, which potentially could be explored as an option to treat MS in the future.

## Additional file

Additional file 1: **Figure S1.** Ablation of RelB in non-immune CNS cells reduces severity of EAE. EAE was induced and clinical scores were recorded for 15 days (*n* = 15–16 mice per group, **Figure S2.** Nestin-driven expression of CRE has no effect on severity of EAE. EAE was induced and clinical scores were recorded for 23 days (*n* = 5–8 mice per group, *p < 0.05, T-test). **Figure S3.** Deletion of RelB in mature oligodendrocytes reduces the severity of EAE. EAE was induced and clinical scores were recorded for 15 days (*n* = 12–17 mice per group, *p < 0.05, T-test). **Table S1.** Infiltration of lymphocytes, CD4+, and CD8+ cells into brains during EAE. EAE was induced, clinical scores were recorded, and flow cytometry was conducted to quantify the indicated cells in the brains. *n* = 4 mice per group. (PDF 240 kb)

## Data Availability

Information regarding the experimental methods used, and the data in this paper are available to scientific communities upon direct contact to the authors. Individual requests for shipment of mice to AAALAC accredited institutions will be honored. An appropriately signed MTA will be required, as well as permission from original sources of RelB^Loxp/LoxP^ mice (NIH).

## Abbreviations

CNS: Central nervous system
EAE: Experimental autoimmune encephalomyelitis
IκBα: Inhibitor of NF-κB alpha
MS: Multiple sclerosis
NF-κB: Nuclear factor kappa B
OPC: Oligodendrocyte precursor cells
p50: Nuclear factor kappa B p50 subunit
p52: Nuclear factor kappa B p52 subunit
p65: Nuclear factor kappa B p65 subunit (RelA)
RelB: Transcription factor RelB
