# Supplementary material for: A detrimental role of RelB in mature oligodendrocytes during experimental acute encephalomyelitis
Source: J Neuroinflammation. 2019 Jul 30;16:161. doi: 10.1186/s12974-019-1548-7 (PMC6664766; doi:10.1186/s12974-019-1548-7)
Supplement: Supplementary file 1 — Figure S1. Ablation of RelB in non-immune CNS cells reduces severity of EAE. EAE was induced and clinical scores were recorded for 15 days (n = 15–16 mice per group, Figure S2. Nestin-driven expression of CRE has no effect on severity of EAE. EAE was induced and clinical scores were recorded for 23 days (n = 5–8 mice per group, *p < 0.05, T-test). Figure S3. Deletion of RelB in mature oligodendrocytes reduces the severity of EAE. EAE was induced and clinical scores were recorded for 15 days (n = 12–17 mice per group, *p < 0.05, T-test). Table S1. Infiltration of lymphocytes, CD4+, and CD8+ cells into brains during EAE. EAE was induced, clinical scores were recorded, and flow cytometry was conducted to quantify the indicated cells in the brains. n = 4 mice per group. (PDF 240 kb) [file 12974_2019_1548_MOESM1_ESM.pdf]

Supplementary Figure 1.

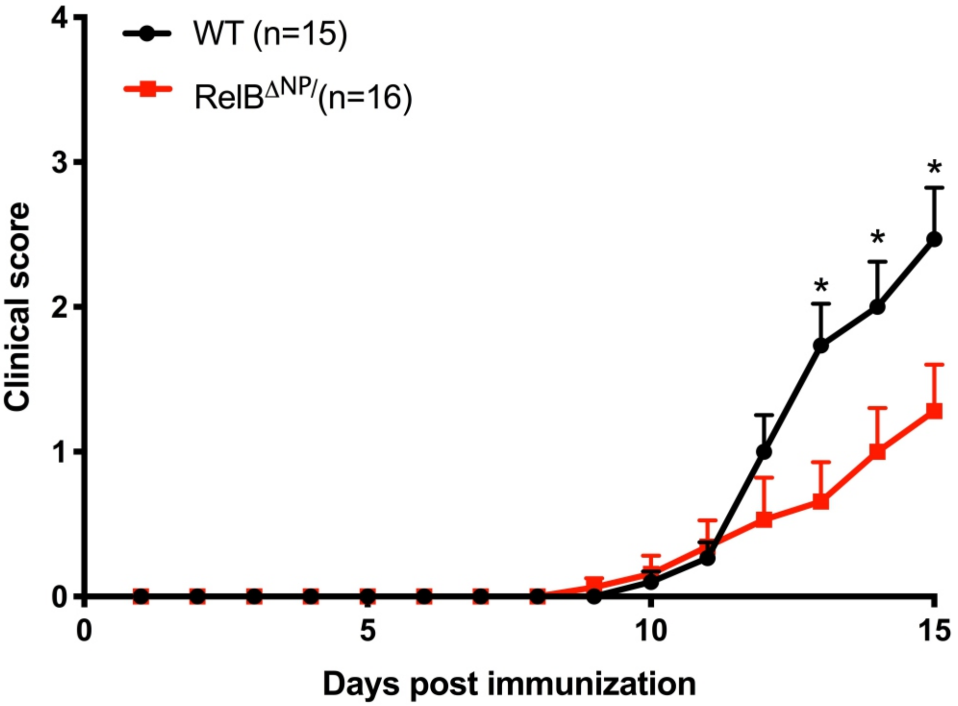

Supplementary Figure 2.

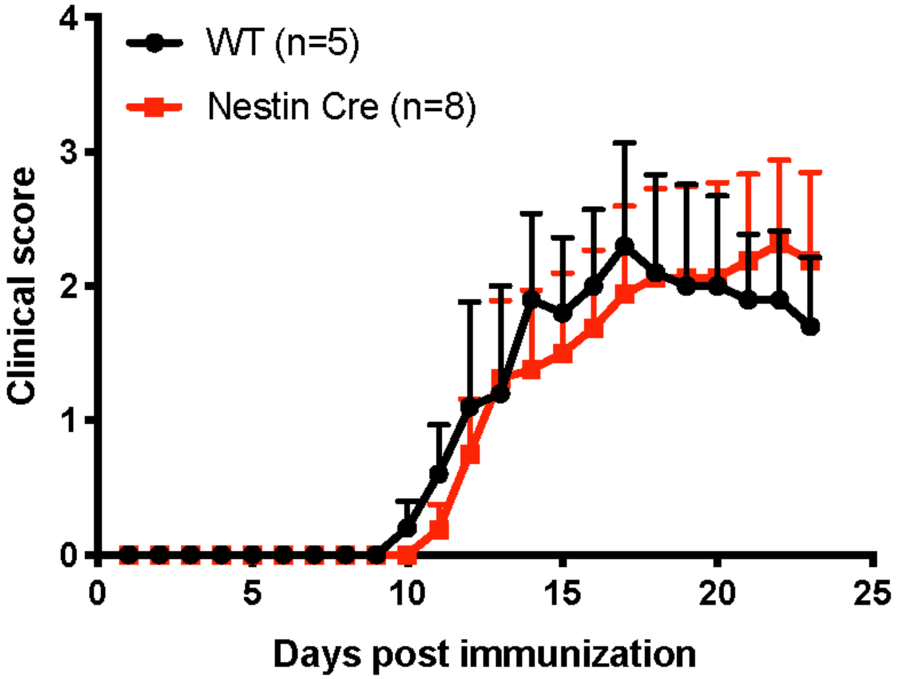

Supplementary Figure 3.

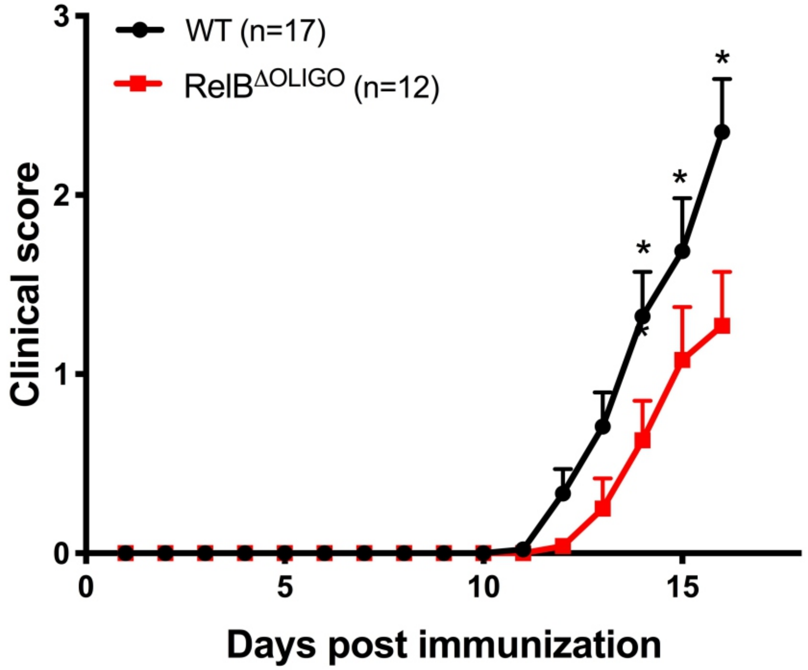

Supplementary Table I

	$CD45^+CD11b^-$	$CD4^+$	$CD8^+$
**WT**	1,239,600±186,800	600,500±70,200	124,900±35,600
**$RelB^{\Delta NP}$**	824,700±172,600	374,000±85,200	105,600±41,600
